# *Notes from the Field:* Fatal *Naegleria fowleri* Meningoencephalitis After Swimming in Hot Spring Water — California, 2018

**DOI:** 10.15585/mmwr.mm6836a3

**Published:** 2019-09-13

**Authors:** Duc J. Vugia, James Richardson, Thomas Tarro, Chairut Vareechon, Pia S. Pannaraj, Elizabeth Traub, Jennifer R. Cope, Sharon Balter

**Affiliations:** ^1^California Department of Public Health; ^2^Inyo County Health and Human Services, Bishop, California; ^3^Children’s Hospital Los Angeles, California; ^4^Los Angeles County Department of Public Health, California; ^5^National Center for Emerging and Zoonotic Infectious Diseases, CDC.

In October 2018, a previously healthy boy was admitted to an intensive care unit at a southern California hospital after experiencing 2 days of headache, vomiting, and fever and 1 day of altered mental status. He was initially treated empirically for bacterial and viral meningitis and subsequently displayed decreased level of consciousness and experienced respiratory failure, requiring intubation and mechanical ventilation. Computed tomography scan of the brain showed diffuse cerebral edema. A wet mount of cerebrospinal fluid obtained by lumbar puncture revealed amebic organisms consistent with *Naegleria* species, and a treatment regimen for *Naegleria* was added, including miltefosine (*1*), which is now commercially available.* The infectious disease clinician notified CDC, which then notified state and local public health. Polymerase chain reaction testing of a cerebrospinal fluid specimen at the Mayo Clinic on hospital day 2 identified *N. fowleri,* a free-living ameba found in warm fresh water that causes primary amebic meningoencephalitis (PAM). The patient’s condition continued to worsen, and he died on hospital day 3.

Family members stated that 12 days before symptom onset the boy had visited a hot spring area in Inyo County, in the Eastern Sierra region of California, where he swam in a natural freshwater pool. This hot spring area is known locally as Hot Ditch, where a stream of warm spring water flows down from the source with several small shallow pools along the course. These untreated (unchlorinated) freshwater pools had been frequented by local residents and visitors for decades without any previous report of PAM.

*N. fowleri* is found in warm freshwater lakes, ponds, and hot springs worldwide, but PAM is rare. In 2014, a Florida boy aged 11 years developed fatal PAM after exposure to a hot spring and river pond in Costa Rica where *N. fowleri* was identified (*2*). In the United States, 145 PAM cases were reported during 1962–2018 (range = 0–8 cases annually); most cases occurred in young males exposed to warm recreational waters during the summer months^†^ (*3*). Infection occurs when water containing *N. fowleri* enters the nose, usually while a person is swimming or diving. The ameba migrates from the nose to the brain along the olfactory nerve through the cribriform plate, destroys brain tissue, and causes cerebral edema. The case fatality rate for PAM exceeds 97% (*3*), and the median time from symptom onset to death is 5 days (*4*). Infection is not transmitted by swallowing contaminated water.

This was the ninth PAM case in California in a patient exposed to warm fresh water and the third in a patient exposed to hot spring water; the first case occurred in 1971 in an adolescent aged 16 years who swam in a desert hot spring in southern California (*5*). In response to this most recent case, Inyo County Health and Human Services issued a press release to inform and warn the public about the potential for *N. fowleri* infection from swimming in the Hot Ditch natural pools and posted warning signs at each of these pools to alert visitors of the risk.

Although contracting PAM is rare after swimming in hot spring water, the potential risk for this disease should be considered, and persons should either refrain from hot spring water–related activities or take actions to prevent spring water from going up the nose (https://www.cdc.gov/parasites/naegleria/swimming.html). Parents should consider this potential risk for their children before exposure to hot spring water.

## Footnotes

* Miltefosine was previously available through CDC. It is now commercially available and can be ordered at https://www.impavido.com/.

† https://www.cdc.gov/parasites/naegleria/infection-sources.html.
